# Eco-anxiety or simply eco-worry? Incremental validity study in a representative Spanish sample

**DOI:** 10.3389/fpsyg.2025.1560024

**Published:** 2025-04-10

**Authors:** María Luisa Vecina, María Alonso-Ferres, Cintia Díaz-Silveira

**Affiliations:** ^1^Faculty of Psychology, Universidad Complutense de Madrid, Madrid, Spain; ^2^Department of Social Psychology, Universidad de Granada, Granada, Spain; ^3^Mind, Brain and Behavior Research Center (CIMCYC), University of Granada, Granada, Spain; ^4^Department of Psychology, Universidad Rey Juan Carlos, Madrid, Spain

**Keywords:** eco-anxiety, eco-worry, climate change, perception, life satisfaction

## Abstract

Scientific literature is keen to promote the study of eco-anxiety despite its current low prevalence and inconsistent relationships with pro-environmental behavior and mental health. In this paper, we analyze in a representative sample of the Spanish population (*N* = 1911) the incremental validity of the eco-worry construct concerning that of eco-anxiety at three levels of environmental commitment: high (environmental activists), medium (people who are not part of any environmental organization but who would like to), and low (people who neither belong to environmental groups nor want to). Our results showed that (1) the environmental activists in our sample did not seem to be eco-anxious but rather eco-worried, and (2) at the three levels of environmental commitment, eco-worry but not eco-anxiety positively mediated the relationship between climate change perception and general willingness for environmental behavior, and eco-worry, but not eco-anxiety, positively connected with life satisfaction through the general willingness to behave pro-environmentally. It is concluded that eco-anxiety does not add anything to the more intuitive and non-pathological concept of eco-worry, except for the alarm signal, which is not at all strategic when the goal is to promote individual pro-environmental behaviors and collective social actions.

## 1 Introduction

Theoretical attempts have been made to distinguish eco-anxiety from eco-concern or eco-worry, insisting that eco-anxiety is also outside pathology (Clayton, 2020; Clayton and Karazsia, 2020; Pihkala, 2020). This is shocking since, intuitively, anxiety is an unpleasant emotional state primarily associated with mental disorders that, of course, no one wants to feel and to which people react negatively as compared to worry or concern (Gregersen et al., 2024). The working definition by the American Psychological Association is quite descriptive in this regard: “A chronic fear of environmental doom.” (Clayton et al., 2017, p. 68). Perhaps to justify this counterintuitive idea, which does not deny that technically, eco-anxiety can be understood from both clinical and non-clinical perspectives (Gino et al., 2012; Weeks et al., 2019), it has been necessary to resort to clarifications such as “practical eco-anxiety,” understood as a specific form of eco-anxiety, that apparently “experienced at the right time and to the right extent, not only reflects well on one’s moral character but can also help advance individual and planetary wellbeing” (Kurth and Pihkala, 2022, p. 1).

In summary, when scientific literature refers to eco-anxiety, it means a multi-faceted phenomenon where “most forms of eco-anxiety appear to be non-clinical.” However, there are cases of “paralyzing forms of eco-anxiety” and also cases of “practical anxiety” that are supposed to lead to the gathering of new information and reassessment of behavior options (Clayton, 2020; Kurth, 2018). In this work, we argue that there may be a problem of conceptual imprecision when referring to different responses with the same generic and negatively connoted label of “eco-anxiety” or under the more specific label of “climate anxiety,” which, according to Pihkala (2020), seems to be the most widely discussed form of eco-anxiety to the extent that some people equate climate anxiety and eco-anxiety.

Numerous research and practical questions arise in this landscape that still have no answers; for instance, what kind of eco-anxiety do existing instruments measure? What does the concept of eco-anxiety add to the concept of eco-worry? Are the people most aware of and committed to environmental problems eco-anxious or eco-worried? Should we try to generate eco-anxiety in, for example, climate deniers to promote pro-environmental behaviors? Is eco-anxiety a valuable concept that motivates social changes?

With the idea of contributing to a better understanding of the concept of eco-anxiety, we study a representative sample of the Spanish population with different levels of pro-environmental commitment (high, medium, and low) regarding a panel of variables necessary to understand what we mean when we talk about eco-anxiety such as climate change perception, eco-worry, general willingness for environmental behavior, and life satisfaction. More specifically, we focus on the incremental validity of the more parsimonious concept of eco-worry, compared to the recent eco-anxiety in the relationship between climate change perception and general willingness for environmental behavior. Additionally, and to elucidate the connections of both concepts with the sphere of mental health, we also test their relationships with life satisfaction through willingness for environmental behavior.

### 1.1 Eco-anxiety, climate anxiety or simply eco-worry?

If we want to attract attention to an urgent problem that is not perceived as such, any prefix accompanied by the term anxiety wins. Maybe, for this reason, Clayton and Karazsia (2020) were among the first to opt for “Climate Change Anxiety.” They delineated the concept as a relatively strong form of anxiety, differentiated from less intense worry that can also be felt irrespective of direct experience with climate change. They developed a 13-item scale to assess how much a person’s everyday functioning is impaired in various domains due to climate change and whether it achieves clinical relevance. The Climate Change Anxiety Scale (CCAS; Clayton and Karazsia, 2020) includes eight items measuring cognitive-emotional impairment (e.g., thoughts about climate change and its effects on concentration, sleep, nightmares, and crying) and five measuring functional impairment, including how climate change concerns affect relationships balance with family and friends or ability to complete work. The authors observed that levels of climate change anxiety were pretty low in their conventional samples and that both climate change anxiety subscales were significantly associated with depression and anxiety and were not related to pro-environmental engagement. These results did not prevent the authors from warning against pathologizing climate anxiety, although their data pointed in that direction.

In addition to these inconsistencies, the CCAS and its rationale present at least three other problems. The first one is its specificity. “Climate Change Anxiety” fell short of the many dimensions of the environmental and climate crisis and is reduced to one of them, the anxiety significantly related to anthropogenic climate change (Hogg et al., 2021). Secondly, as noted by Wullenkord et al. (2021), the CCAS assesses various impairments stemming from climate change rather than the emotional experience of climate change anxiety. Thirdly, it presents “some ambiguity about its factor structure” (Cruz and High, 2022, p. 1; see also Innocenti et al., 2021) and fails to “capture gradations and degrees of severity of climate anxiety” (Wullenkord et al., 2021, p. 16).

The Hogg Eco-Anxiety Scale addressed some of these concerns (HEAS-13; Hogg et al., 2021). It describes any anxiety that is related to the global ecological crisis and the state of the planetary ecosystems; it also includes an affective symptoms subscale, but it does not achieve either capturing degrees of severity of eco-anxiety. It seems that both the CCAS and the HEAS-13 assess more severe responses to anxiety, which suggests that a considerable number of items from both instruments are difficult to endorse unless a person is more strongly eco-anxious (Wullenkord et al., 2021).

With this empirical evidence, nothing would have prevented reserving the terms “Eco-anxiety” and “Climate Anxiety” only for the strongest anxiety responses and only for a minority percentage of people who probably would benefit from clinical treatments. However, this is not what seems to be happening. Most researchers follow Pihkala (2020, p. 12) when he states that these terms should also include less severe worry and fear for several reasons: “First since scholars warn against pathologizing eco-anxiety and climate anxiety, such anxiety should be defined in a wide manner. Second, anxiety itself is such a multidimensional phenomenon that it would be rather narrow to restrict eco-anxiety and climate anxiety only to stronger anxiety symptoms.” These reasonable reasons put us in the starting box: Why is the concept of eco-anxiety necessary over the more intuitive concept of eco-worry? Actually, nobody doubts that eco-worry is not necessarily pathological and motivates pro-environmental behaviors (Böhm et al., 2023; Bouman et al., 2020; Kurt and Akdur, 2024; Parmentier et al., 2024; Verplanken et al., 2020).

If as stated by Pihkala (2020, p. 4), “the typical form of eco-anxiety is sometimes seen more as related to worry and sometimes more as related to strong anxiety” what we would need is at least two different terms and two different instruments, one to refer to the less intense emotion of worry and other to the more intense anxiety that keeps people awake and ruminating often. We would say that in a continuum of intensity where worry ends, anxiety begins (Lutz et al., 2023). When anxiety begins, we will be clearly entering the realm of the potentially pathological, so we will not expect to find strong connections with pro-environmental behaviors or psychological well-being. Of course, we will not take eco-anxiety as a reflection of a person’s moral character. The tendency to replace adjectives such as nervous, stressed, and sad with diagnoses that have clear and often severe consequences for people’s lives (e.g., anxiety, panic, and depression) is a mockery of those who are really struggling (Appelkvist, 2020) and likely unhelpful in motivating change in those who are not.

### 1.2 Objectives and hypotheses

**Figure 1:**
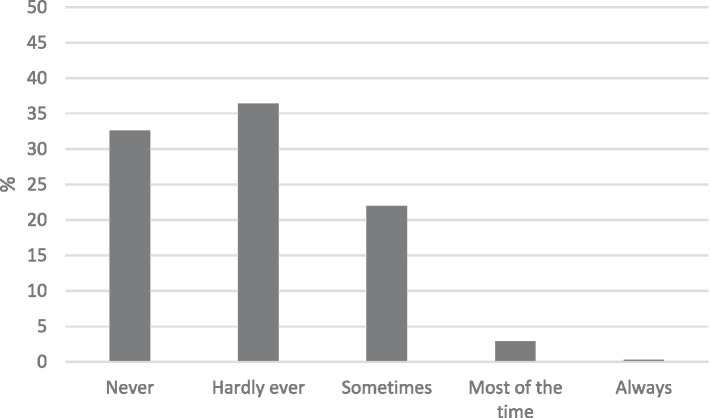
Percentages of eco-anxiety in the representative Spanish sample (*N* = 1911).

In order to help elucidate whether the concept of eco-anxiety is usurping the place of eco-worry in the continuum of possible emotional responses to the environmental and climate crisis, we will analyze in a representative sample of the Spanish population the incremental validity of the eco-worry construct concerning that of eco-anxiety, first, in a role of mediators between the climate change perception and the general willingness for environment behavior, and second, in a subsequent serial mediation which connect such willingness for environment behavior with life satisfaction (see Figure 1; Model 0). Since individuals vary widely in their value on environmental issues and the severity with which they perceive the environmental crisis (Helm et al., 2018), their associated emotional and behavioral responses may also do it. So, we will test the model at three levels of environmental commitment: high (environmental activists), medium (people who are not part of any environmental organization but would like to), and low (people who neither belong to environmental groups nor want to).

Since the scientific literature seems to be keen to promote the study of eco-anxiety despite its current low prevalence and inconsistent relationships with pro-environmental behaviors (Clayton and Karazsia, 2020; Heeren et al., 2022; Innocenti et al., 2021; Kühner et al., 2024; Leite et al., 2023; Stanley et al., 2021) and mental health (Gago et al., 2024; Hogg et al., 2021; Reyes et al., 2023; Schwartz et al., 2023), we will use the aforementioned differentiation between levels of environmental commitment to try to recreate the most favorable conditions for experiencing eco-anxiety. According to Clayton (2020), “Climate anxiety is not evenly distributed; it is more common among those who care about environmental issues” (p. 12). So, we should find the ideal conditions for eco-anxiety among environmental activists who are most aware of and committed to environmental issues. Our specific hypotheses are the following:

*H1*: We expect to find the highest levels of perceived severity of climate change in the activist group and, therefore, also the highest levels of eco-anxiety, eco-worry, and general intention for environmental behavior, and all this at similar levels of life satisfaction since it seems that environmentally friendly lifestyle changes need not entail reductions in individuals’ well-being (Prinzing et al., 2024; Zawadzki et al., 2020). We also expect that, as it is found but ignored (Clayton and Karazsia, 2020; Cruz and High, 2021; Innocenti et al., 2021; Hogg et al., 2021; Leite et al., 2023; Whitmarsh et al., 2022; Wullenkord et al., 2021), levels of eco-anxiety will be low (below the midpoint of the scale) and lower than those of eco-worry, even in the activist group.

*H2*: We expect that eco-worry, measured as recently proposed again by Parmentier et al. (2024), will be a better mediator in the relationship between climate change perception and willingness for environmental behavior than eco-anxiety, measured with the HEAS-13 (Hogg et al., 2021), at any of the three levels of environmental commitment. Additionally, eco-worry will connect to life satisfaction better than eco-anxiety through the general willingness for environmental behavior.

## 2 Method

### 2.1 Participants and procedure

Our sample was collected from a market research company with online panelists. A stratified random sample was drawn for each Spanish region, and quotas were set for sociodemographic variables. The sample was representative of the Spanish population in terms of gender, age, and place of residence.

The total sample comprised 1911 participants (*M*_age_ = 48.13, SD = 15.23, range from 18 to 75). Fifty-point-seven percent were women, and 49.3% were men. A total of 8.6% of the participants completed elementary school, 20% high school, 31.1% vocational training, 28.6% had a bachelor’s degree, and 11.7% had a master’s degree. Regarding their levels of environmental commitment, 5.6% of the participants indicated to be highly environmentally committed (environmental activists, *N* = 107). These individuals were defined as those who actively participated in time-demanding activities in addition to paying membership fees to an environmental organization. They all specified the organization to which they belonged and described their activities. 43.5% indicated that they did not belong to any environmental organization but would very much like to (medium commitment group, *N* = 832), and 50.9% were classified in the group with low commitment (*N* = 972). They neither belonged to environmental organizations nor wanted to.

Participation in the study was voluntary, and data were saved and processed anonymously. All participants provided informed consent about participation and publication. The data collection was approved by the Ethics Committee of [anonymized] (protocol code 0407202327123, September 2023). Code and password-protected data are available at Open Science Framework: https://osf.io/6qn8y.

A sensitive power analysis was conducted using ANOVA: fixed effects, special, main effects, and interactions in G*Power (Faul et al., 2009) to determine our ability to detect the effect of the environmental commitment level (low, medium, or high) on eco-anxiety, eco-worry, climate change perceptions, general willingness for environmental behavior, and satisfaction with life. Considering our sample (*N* = 1911, *α* = 0.05), the sensitivity analysis suggests that effect sizes of *f* ≥ 0.071 are necessary to produce power at the 0.80 level.

### 2.2 Measures

#### 2.2.1 Eco-worry scale (EWS)

This 5-item scale from Parmentier et al. (2024) assesses the frequency and relative intensity of worrying thoughts related to environmental issues in general, including a specific item addressing apprehension about one’s impact on the planet (e.g., “I am concerned about the impact of my behaviors and lifestyle on the Earth.,” “Climate change makes me worry about my future and that of the people I care about” and “I worry about the environmental crisis more than other people”). Participants rated all items on a 5-point scale, ranging from “not at all” to “extremely,” except for item 1 (“How often do you have thoughts about environmental issues that concern you?”), which ranged from “never” to “almost always.” Cronbach’s alpha coefficient of 0.86.

#### 2.2.2 The Hogg eco-anxiety scale (HEAS-13)

The Hoog Eco-Anxiety Scale (HEAS-13) from Hogg et al. (2021) measures anxiety in response to the global environmental crisis through four underlying factors: affective symptoms (4 items), behavioral symptoms (3 items), negative emotionality (3 items), and rumination (3 items). A 6-month time frame instead of a two-week was used in the instructions to ensure the stability of the measurement under the most favorable conditions for obtaining high eco-anxiety: “Over the last 6 months, how often have you been bothered by the following problems when thinking about climate change and other global environmental conditions (e.g., global warming, ecological degradation, resource depletion, species extinction, ozone hole, pollution of the oceans, deforestation)? Some example items are listed as follows: “Worrying too much” (affective symptom), “Unable to stop thinking about past events related to climate change” (rumination), “Difficulty working and/or studying” (behavioral symptom), and “Feeling anxious about the impact of your behaviors on the Earth” (negative emotionality). It was used a 5-point Likert scale ranging from 1 (never) to 5 (always). Cronbach’s alpha was 0.96.

#### 2.2.3 Climate change perceptions scale

Climate change perceptions scale from Van Valkengoed et al. (2021) measures five dimensions of climate change: the perceived reality, human causes, negative consequences, spatial proximity, and the temporal distance of its implications. We used the short version of five items (1 = strongly disagree; 5 = strongly agree). The items were as follows: ‘I believe that climate change is real’ (reality); ‘The main causes of climate change are human activities’ (causes); ‘Climate change will bring about serious negative consequences’ (valence of consequences); ‘My local area will be influenced by climate change’ (spatial distance of consequences); and ‘It will be a long time before the consequences of climate change are felt’ (temporal distance of consequences, R). Cronbach’s alpha was 0.82.

#### 2.2.4 General willingness for environmental behavior scale (GWEBS; Vecina et al., 2024)

It assesses the general willingness to do or not (degrowth and reduced consumption), accept social restrictions, and ultimately “do your bit” for the environment. The items were as follows: “Assess the likelihood that you will incorporate new environmental actions into your daily life over the next year” (from 1 = never to 5 = always); “Within my possibilities, I wish to do my bit to stop the environmental crisis”; “I am willing to voluntarily decrease (consume less matter and energy)”; and “I am willing to accept the social restrictions that are necessary to improve the environmental situation” (from 1 = strongly disagree to 5 = strongly agree). Cronbach’s alpha was 0.82.

#### 2.2.5 Satisfaction with life

The 5-item Satisfaction with Life Scale (Diener et al., 1985) was used. Participants indicated the extent to which they are satisfied with their life (e.g., “In most ways, my life is close to my ideal”; “I am satisfied with my life”). A 5-point Likert scale ranging 1 (strongly disagree) to 5 (strongly agree) was used. Cronbach’s alpha was 0.87.

### 2.3 Results

#### 2.3.1 Prevalence rates of eco-anxiety and eco-worry

**Figure 2:**
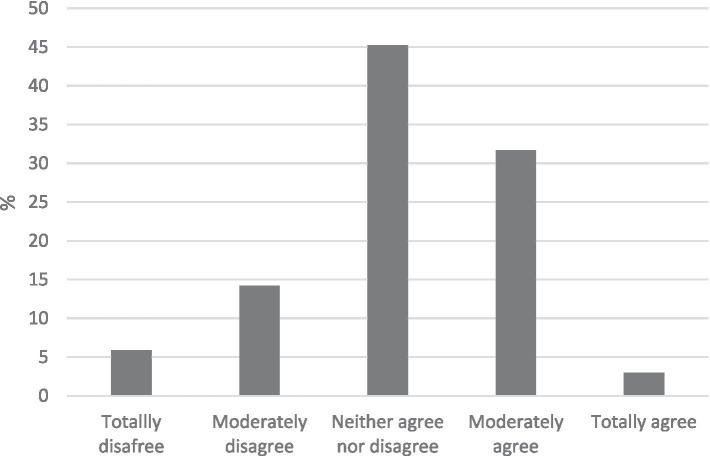
Percentages of eco-worry.

The results indicated that a significant portion of the participants (32.6 and 36.4%) reported never or hardly ever felt eco-anxiety when reflecting on climate change and other global environmental issues in the last 6 months (Figure 1). Conversely, regarding eco-worry, 45.2 and 31.7% of respondents expressed being moderately or very worried about climate change (Figure 2).

In order to determine the prevalence, responses were categorized into ‘mild’ (1.00 ≤ M ≤ 2.33), ‘moderate’ (2.34 ≤ M ≤ 3.66), and ‘severe’ levels (3.67 ≤ M ≤ 5.00), following established practices for interpreting Likert scale data in the absence of standardized cut-off scores (e.g., De Vaus, 2002; Whitmarsh et al., 2022). Prevalence rates were expressed as percentages for each category and further analyzed by the level of environmental commitment (Table 1). Overall, eco-anxiety levels were lower (*M* = 2.3; SD = 0.87) compared to eco-worry (*M* = 3.51; SD = 0.88). Notably, 51.8% of respondents exhibited mild eco-anxiety, while only 8.9% reported mild levels of eco-worry. Conversely, only 6.1% of respondents exhibited severe eco-anxiety, while 44.6% reported severe levels of eco-worry.

**Table 1:** Prevalence rates of eco-anxiety and eco-worry.

		Prevalence rates (%)						*M* (SD)	
		Mild		Moderate		Severe			
		Eco-anxiety	Eco-worry	Eco-anxiety	Eco-worry	Eco-anxiety	Eco-worry	Eco-anxiety	Eco-worry
	All	51.8	8.9	42.1	46.4	6.1	44.6	2.3 (0.87)	3.51 (0.88)
Environment commitment	Low	66.7	16.4	30.8	60.3	2.6	23.4	2.01 (0.83)	3.10 (0.88)
	Medium	37.9	1.2	54.4	33.9	7.7	64.9	2.56 (0.79)	3.91 (0.63)
	High	25	0.9	50	17.8	25	81.3	2.94 (0.87)	4.12 (0.68)

In line with our hypothesis, it can be observed that the mean score in eco-anxiety was low in all three groups based on their level of environmental commitment, even though the time window in our study was extended from 2 weeks to 6 months. More specifically, the environmental activist’s levels of eco-anxiety were also low and lower than those of eco-worry. In other words, the frequency with which participants reported experiencing signs of eco-anxiety in the past 6 months did not exceed the scale’s midpoint (3), even in the highly committed environmental activist group.

Subsequently, to examine whether there were significant differences between eco-worry and eco-anxiety within each group, we conducted paired-samples *t*-tests separately for each level of environmental commitment. Among individuals with low environmental commitment, the results showed a significant difference between eco-worry (*M* = 3.10, *SD* = 0.88) and eco-anxiety (*M* = 2.01, *SD* = 0.83), *t*(971) = 37.11, *p* < 0.001. In the medium environmental commitment group, a significant difference was also found between eco-worry (*M* = 3.91, *SD* = 0.63) and eco-anxiety (*M* = 2.56, *SD* = 0.79), *t*(831) = 46.27, *p* < 0.001. Similarly, among individuals with high environmental commitment, the analysis revealed a statistically significant difference, with higher levels of eco-worry (*M* = 4.12, *SD* = 0.68) compared to eco-anxiety (*M* = 2.94, *SD* = 0.87), *t*(106) = 11.70, *p* < 0.001. These findings suggest that while eco-worry and eco-anxiety are conceptually related, their intensity varies in each level of environmental commitment.

#### 2.3.2 Effects of the environmental commitment level on study variables

A one-way ANOVA was conducted to compare the effect of the environmental commitment level (low, medium, or high) on eco-anxiety, eco-worry, climate change perceptions, general willingness for environmental behavior, and satisfaction with life. The analysis revealed a significant effect of the level of environmental commitment on eco-anxiety, 𝐹(2,1907) = 136.98, 𝑝 < 0.001, 𝜂^2^ = 0.13; eco-worry 𝐹(2,1907) = 287.91, 𝑝 < 0.001, 𝜂^2^ = 0.23; climate change perceptions, 𝐹(2,1907) = 80.75, 𝑝 < 0.001, 𝜂^2^ = 0.08; and general willingness for environmental behavior, 𝐹(2,1907) = 203.58, 𝑝 < 0.001, 𝜂^2^ = 0.20. No significant effect of the level of environmental commitment on satisfaction with life were found, (2,1907) = 2.12, 𝑝 = 0.121, 𝜂^2^ = 0.00.

*Post hoc* comparisons using the Tukey HSD test (see Table 2) revealed that the highly environmentally committed group scored significantly higher in eco-anxiety compared to the moderately committed (𝑝 < 0.001) and low-commitment groups (𝑝 < 0.001). Additionally, the moderately committed group displayed significantly higher eco-anxiety scores than the low-commitment group (𝑝 < 0.001). Regarding eco-worry, the highly environmentally committed group also scored significantly higher compared to the moderately committed (𝑝 = 0.024) and low-commitment groups (𝑝 < 0.001). Likewise, the moderately committed group displayed significantly higher scores than the low-commitment group (𝑝 < 0.001). Finally, concerning climate change perceptions and the general willingness for environmental behavior, results showed that the highly environmentally committed group scored significantly higher compared to the low-commitment groups (𝑝s < 0.001) while the moderately committed group displayed significantly higher scores than the low-commitment group (𝑝s < 0.001). However, no significant differences were found between the high and moderate environmentally committed groups in climate change perception (𝑝 = 0.629) and the general willingness for environmental behavior (𝑝 = 0.243).

**Table 2:** *Post hoc* analyses.

	Level of environmental commitment *M* (SD)		
	Low	Medium	High
Eco-anxiety	2.01 (0.83)	2.56 (0.79)	2.94 (0.87)
Eco-worry	3.10 (0.88)	3.91 (0.63)	4.12 (0.68)
Climate Change Perception	3.83 (0.90)	4.31 (0.68)	4.23 (0.75)
General Willingness for environmental Behavior	3.25 (0.96)	4.05 (0.67)	4.19 (0.72)
Satisfaction with life	3.33 (0.89)	3.39 (0.83)	3.48 (0.83)

In line with our hypothesis, the environmental activist group reported having (1) the most serious perception of the climate situation together with the group with medium environmental commitment, (2) the highest level of eco-anxiety and eco-worry, and (3) the highest willingness to act pro-environmentally together with the group with medium environmental commitment. Life satisfaction was similar in the three groups.

#### 2.3.3 Correlational analyses

Table 3 presents the correlation coefficients for the study variables across groups categorized by their level of environmental commitment. All correlations were below 0.70, indicating no concerns about multicollinearity. The results of the groups with low and medium environmental commitment followed a similar pattern: eco-anxiety was positively associated with eco-worry, climate change perception, and general willingness for environmental commitment, but no association was found with life satisfaction. It is important to note that while the association between eco-worry and eco-anxiety was moderate, the association with climate change perception was small, as was the association with general willingness for environmental behavior in the medium environmental commitment group. On the other hand, Eco-worry showed strong positive associations with climate change perception, general willingness for environmental behavior, and life satisfaction. Additionally, significant positive and strong correlations were found between climate change perception and general willingness for environmental behavior, as well as between the latter and life satisfaction. However, the effect size for these correlations was small. In contrast, the environmental activist group exhibited a different pattern. Eco-anxiety was not associated with any study variable, whereas eco-worry showed strong positive correlations with climate change perception and general willingness to engage in environmental behavior. It is important to note the small number of subjects in the activist group, which may explain why some small correlations are not significant in this group due to the sample size.

**Table 3:** Correlations among the variables based on individuals’ level of environmental commitment.

	Level of environmental commitment														
	Low					Medium					High				
	1	2	3	4	5	1	2	3	4	5	1	2	3	4	5
1 Eco-anxiety	—					—					—				
2 Eco-worry	0.43^***^	—				0.31^***^	—				0.11	—			
3 CCP	0.10^***^	0.59^***^	—			0.11^***^	0.49^***^	—			−0.14	0.58^***^	—		
4 GWEB	0.27^***^	0.62^***^	0.49^***^	—		0.08^*^	0.47^***^	0.44^***^	—		0.09	0.56^***^	0.62^***^	—	
5 Life satisfaction	−0.01	0.05	−0.07^*^	0.13^***^	—	0.01	0.14^***^	−0.04	0.15^***^	—	0.12	0.12	−0.02	0.14	—

CCP, Climate Change Perception; GWEB, General Willingness for Environmental Behavior; ***p < 0.001.

#### 2.3.4 Predicting eco-anxiety and eco-worry from climate change perception

A MANOVA was conducted to explore whether climate change perception predicts eco-anxiety and eco-worry across the three levels of environmental commitment. The MANOVA showed a significant multivariate effect for the low-committed participants, Wilks’ Lambda = 0.586, *F*(40, 1738) = 13.31, *p* < 0.001. Climate change perception significantly predicted eco-anxiety, *F*(1, 890) = 2.04, *p* = 0.005, *η*^2^ = 0.045, and strongly predicted eco-worry, *F*(1, 890) = 25.64, *p* < 0.001, *η*^2^ = 0.371.

The MANOVA indicated a significant multivariate effect for the medium-committed participants, Wilks’ Lambda = 0.62, *F*(36, 1,508) = 11.316, *p* < 0.001. Climate change perception notably predicted eco-anxiety, *F*(1, 755) = 2.14, *p* = 0.004, *η*^2^ = 0.049, and strongly predicted eco-worry, *F*(1, 755) = 17.92, *p* < 0.001, *η*^2^ = 0.299.

Similarly, a significant multivariate effect was observed for the high-committed participants, Wilks’ Lambda = 0.37, *F*(28, 194) = 4.50, *p* < 0.001. Climate change perception significantly predicted eco-anxiety, *F*(1, 98) = 1.86, *p* = 0.041, *η*^2^ = 0.210 (21.0% of variance), and robustly predicted eco-worry, *F*(1, 98) = 7.73, *p* < 0.001, *η*^2^ = 0.525 (52.5% of variance). These results indicate that climate change perception has a much stronger association with eco-worry than eco-anxiety for high-committed participants.

#### 2.3.5 Incremental validity of eco-worry above eco-anxiety predicting general willingness for environmental behavior

We examined whether eco-worry provides a unique predictive ability beyond eco-anxiety in explaining general willingness for environmental behavior. Hierarchical regression analyses were conducted for each group based on their environmental commitment level. In Step 1, the general willingness for environmental behavior was regressed on eco-anxiety. In Step 2, eco-worry was added to the model.

Results showed that eco-anxiety significantly predicted general willingness for environmental behavior, explaining 7% of the variance for the low environmental commitment group [𝐹_Change (1, 971)_ = 78.82, 𝑝 < 0.001]. It accounted for 1% for the medium environmental commitment group [𝐹_Change (1,830)_ = 4.47, 𝑝 = 0.035] and 0% for the environmental activist group [𝐹_Change (1,106)_ = 0.01, 𝑝 = 0.363]. Adding eco-worry significantly improved the model, explaining an additional 31.3% of the variance in the group with low environmental commitment [𝐹_Change (1,971)_ = 495.13, 𝑝 < 0.001], 22.1% in the group with medium environmental commitment [𝐹_Change (1,830)_ = 237.36, 𝑝 < 0.001], and 30.2% in the environmental activist group [𝐹_Change (1,106)_ = 47.02, 𝑝 < 0.001]. These findings, summarized in Table 4, highlight that eco-worry provides significant additional predictive value for general willingness for environmental behavior, over and above eco-anxiety.

**Table 4:** Hierarchical regression analysis predicting general willingness for environmental behavior.

	Level of environmental commitment		
	Low	Medium	High
Predictors	ß	ß	ß
Model 1			
Eco-anxiety	0.27^***^	0.07^*^	0.09
Model 2			
Eco-anxiety	0.01	−0.08^**^	0.03
Eco-worry	0.62^***^	0.50^***^	0.56^***^
Total *R*^2^	0.39	0.23	0.31

Standardized regression coefficients are reported. Bootstrap sample size: 5,000. *p < 0.05, **p < 0.01, ***p < 0.001.

#### 2.3.6 Indirect effect of climate change perception on life satisfaction based on rates of eco-anxiety, eco-worry, and general willingness for environmental behavior

**Figure 3:**
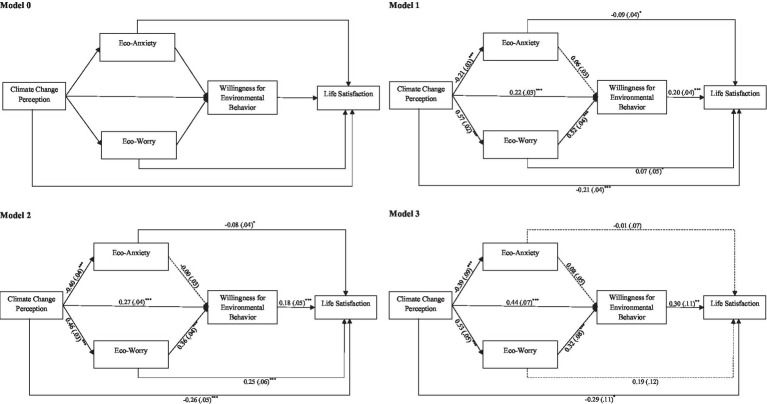
Conceptual model showing the indirect effect of multiple steps between Climate Change Perception and Life Satisfaction via Eco-anxiety, Eco-worry, and Willingness for Environmental Behavior, based on their level of environmental commitment: Low (Model 1), Medium (Model 2), and High (Model 3). Standardized beta coefficients reported with standard errors within parentheses. *p < 0.05, ** *p* < 0.01, ***p < 0.001.

After examining the effect of climate change perception on eco-anxiety and eco-worry, as well as the incremental validity of eco-worry over eco-anxiety in predicting general willingness for environmental behavior, serial mediation analyses were conducted using PROCESS (Model 6; Hayes, 2013). These analyses explored the indirect standardized effects of climate change perception on life satisfaction through eco-anxiety, eco-worry, and general willingness for environmental behavior within each environmental commitment group (see Figure 3, Model 0). Climate change perception was entered as the predictor variable (X), and satisfaction with life as the outcome variable (Y). Eco-anxiety (M1), eco-worry (M2), and general willingness for environmental behavior (M3) were included as mediators. Following Hayes (2013) methodology, bias-corrected confidence intervals for the indirect effects were estimated using 10,000 bootstrap samples. A confidence interval (CI) excluding 0 indicated a statistically significant indirect effect.

As shown in Figure 3 (Model 1), several key relationships were identified for participants with low environmental commitment. First, climate change perception was positively associated with eco-anxiety and eco-worry, explaining 1 and 48% of the variance, respectively. Second, while eco-anxiety was unrelated to general willingness for environmental behavior, eco-worry positive and significantly predicted it, explaining 42% of its variance after controlling climate change perception. Third, the general willingness for environmental behavior positively predicted life satisfaction, accounting for 5% of its variance after adjusting for the other variables. Furthermore, climate change perception negatively predicted life satisfaction (*total effect: b* = −0.07, *SE* = 0.03, *p* = 0.036, 95% CI [−0.13, −0.01]), an effect that remained significant after controlling for all variables (*direct effect: b* = −0.21, *SE* = 0.04, *p* < 0.001, 95% CI [−0.29, −0.13]). Finally, an indirect effect of climate change perception on life satisfaction emerged via eco-worry and the general willingness for environmental behavior (*b* = 0.06, *SE* = 0.01, 95% CI [0.04, 0.09]). No such indirect effect was found through eco-anxiety (*b* = 0.00, *SE* = 0.00, 95% CI [−0.01, 0.00]). This pattern suggests that for the group with low environmental commitment (which is not and does not want to be part of environmental organizations), perception of climate change was related to higher satisfaction with life primarily through increased eco-worry, which, in turn, enhanced their general willingness to behave pro-environmentally.

For participants with medium environmental commitment (Model 2), a similar pattern emerged regarding the role of eco-anxiety and eco-worry. First, climate change perception was negatively associated with eco-anxiety and positively with eco-worry, explaining 1 and 38% of the variance, respectively. Second, eco-anxiety was unrelated to general willingness for environmental behavior, while eco-worry significantly predicted it, accounting for 28% of its variance. Third, general willingness for environmental behavior positively predicted life satisfaction (5% of variance). Furthermore, although climate change perception did not predict life satisfaction (*total effect: b* = −0.05, *SE* = 0.04, *p* = 0.250, 95% CI [−0.14, 0.04]), a significant association emerged after controlling for climate change perception, eco-anxiety, eco-worry, and general willingness for environmental behavior (*direct effect: b* = −0.26, *SE* = 0.05, *p* < 0.001, 95% CI [−0.37, −0.15]). Finally, an indirect effect of climate change perception on life satisfaction occurred via eco-worry and general willingness for environmental behavior (*b* = 0.02, *SE* = 0.01, 95% CI [0.01, 0.04]), highlighting their mediating role. In contrast, no effect was found through eco-anxiety (*b* = 0.00, *SE* = 0.00, 95% CI [−0.01, 0.005]).

Finally, for participants with a high level of environmental commitment (Model 3), climate change perception was negatively associated with eco-anxiety and positively with eco-worry, explaining 9 and 39% of the variance, respectively. Second, while eco-anxiety did not show any significant association with general willingness for environmental behavior, eco-worry emerged as a significant predictor, explaining 47% of its variance after controlling for climate change perception. Third, general willingness for environmental behavior positively influenced life satisfaction, accounting for 8% of its variance after adjusting for climate change perception, eco-anxiety, and eco-worry. Although climate change perception did not directly predict life satisfaction (*total effect: b* = −0.00, *SE* = 0.08, *p* = 0.962, 95%CI [−0.16, 0.15]), a significant association emerged when accounting for all the variables (*direct effect: b =* −0.29, *SE* = 0.11, *p* = 0.011, 95%CI [−0.07, −0.27]). Finally, an indirect effect of climate change perception on life satisfaction was observed via eco-worry and the general willingness for environmental behavior (*b* = 0.05, *SE* = 0.03, 95%CI [0.01, 0.10]). No similar indirect effect was found through eco-anxiety (*b* = −0.01, *SE* = 0.01, 95%CI [−0.03, 0.00]). This pattern again highlights that climate change perception is related to increased life satisfaction through increased eco-worry and, subsequently, higher general willingness for environmental behavior.

In line with our second hypothesis, eco-worry—rather than eco-anxiety—played a pivotal role in mediating the relationship between climate change perception, willingness to act pro-environmentally, and life satisfaction, regardless of the level of environmental commitment (low, medium, or high). A stronger perception of the severity of climate change significantly increased eco-worry, which, in turn, enhanced the willingness to engage in pro-environmental behavior and, ultimately, life satisfaction. It is important to note that although the indirect effect was statistically significant across all levels of environmental commitment, its size was very small.

## 3 Discussion

The concept of eco-anxiety is far from being clear, and further theoretical development has been required to advance conceptual understanding of eco-anxiety (Coffey et al., 2021). However, it is indisputable that eco-anxiety is a topic that attracts attention in current research despite some inconsistencies that also require attention (Innocenti et al., 2021; Rodríguez Quiroga et al., 2024; Vecina et al., 2024; Whitmarsh et al., 2022; Wullenkord et al., 2021). In this paper, we focus on them and try to elucidate where eco-anxiety, measured by the HEAS-13 (Hogg et al., 2021), should be placed on the hypothetical continuum of responses to the environmental crisis.

The dominant literature maintains that eco-anxiety is one of the possible responses to the climate crisis, which is different and more serious than eco-worry but which is not pathological either. It could be said that the space that this understanding of eco-anxiety leaves in such a hypothetical continuum is narrow, subtle, and even incapable of housing its own continuum of responses. In this respect, there are several problems with the concept of eco-anxiety. First, the most frequently used instruments, the CCAS and the HEAS-13 seem to be unable to reflect high levels of eco-anxiety at the population level, so the population average seems to be consistently relatively low, mild, insignificant (Clayton and Karazsia, 2020; Cruz and High, 2021; Hogg et al., 2021; Leite et al., 2023; Whitmarsh et al., 2022; Wullenkord et al., 2021). Second, the relationships with pro-environmental behaviors seem to be inconsistently variable from null to medium-sized (Clayton and Karazsia, 2020; Heeren et al., 2022; Innocenti et al., 2021; Leite et al., 2023; Lutz et al., 2023; Stanley et al., 2021; Verplanken et al., 2020; Wullenkord et al., 2021). Third, the relationships with mental health, although variable, lean toward positive relationships with several mental disorders of varying magnitude (Gago et al., 2024; Hogg et al., 2021; Prinzing et al., 2024; Reyes et al., 2023; Schwartz et al., 2023; Zawadzki et al., 2020).

To justify the intensive use that is currently being made of eco-anxiety, it would be necessary to require: (1) that a significant percentage of the population has eco-anxiety to a high or very high degree, (2) that the concept of eco-anxiety provides something added compared to other similar concepts, and (3) that its use proves to be helpful in motivating the pertinent changes that the system needs to face the environmental and climate crisis. So, in this paper, we used a representative Spanish sample and tried to find eco-anxiety where according Clayton (2020) it was likely to be, that is, among environmental activists who are aware of the magnitude of the crisis and who also shape their lives to have a lesser impact in the face of a constantly worsening planetary situation. However, we did not find it. We also compared the added value that the concept of eco-anxiety brings to that of eco-worry in a serial mediation model that illustrates the relationships of both concepts with climate change perception, willingness to behave pro-environmentally, and satisfaction with life. However, we did not find any either.

Specifically, our results showed, on the one hand, that environmental activists reported having a profound perception of the climate situation, higher levels of eco-anxiety and eco-worry than any other group, and elevated willingness to act pro-environmentally. All this is achieved at levels of satisfaction with their lives similar to those of other population groups with less environmental commitment. However, their absolute levels of eco-anxiety were low and lower than those of eco-worry. On the other hand, results showed that at all levels of commitment to the environment, their eco-worry, but not their eco-anxiety, connected the perception of the situation as a serious problem caused by humans with the necessary general willingness to behave pro-environmentally, and that said willingness to behave pro-environmentally mediated the subsequent positive relationship between eco-worry and satisfaction with life.

These results are in line with those showing that (1) eco-worry is the most frequently studied climate-related emotion and the most intensely experienced (Böhm et al., 2023; Bouman et al., 2020; Gregersen et al., 2024; Martin et al., 2023; van der Linden, 2017; Stewart, 2021); (2) eco-worry plays a constructive role with respect to the commitment to pro-environmental behaviors (van der Linden, 2017), with no additional contribution from the climate anxiety reaction involving impairments (Parmentier et al., 2024), and (3) eco-worry is the intuitive alternative that already existed when the contagious interest in eco-anxiety arose (Böhm et al., 2023; Sundblad et al., 2014; Verplanken and Roy, 2013). The one that, in addition, is clearly functional and promotable.

So, we can conclude that people who are most aware of and committed to environmental issues are more eco-worried than eco-anxious, and their eco-anxiety does not seem to connect their perception of the environmental crisis with their willingness to take action. Nor does it connect with their life satisfaction through the willingness to act pro-environmentally. The same results were obtained in the other two groups with less environmental commitment. Together, they all come to indicate that it is necessary to critically review the concept of eco-anxiety since it does not seem to contribute anything added to the more intuitive and clearly non-pathological concept of eco-worry, at least in our sample, and as long as the essential objective is to activate the general population in the fight against the multiple crises that the planet faces.

### 3.1 Limitations

We cannot conclude from our cross-sectional study that eco-worry is an explanatory variable of the willingness to behave pro-environmentally and less satisfaction with life. Moving beyond correlational studies is essential to understand how eco-worry and eco-anxiety evolve and circumstances. Despite that we provide empirical evidence that eco-anxiety works comparatively poorly in the proposed network of relationships and in the selected Spanish sample. Furthermore, while the indirect effects in the mediation models are significant, their size is likely very small.

Regarding the sample, and despite its representativeness, the generalizability of the results may be limited to the social and cultural context of Spain and the controlled variables. While quotas ensured representativeness for gender, age, and place of residence, other variables like income or education level may not be equally balanced. Since the sample was collected from an online panel, it might exclude individuals without internet access or those not engaged with digital platforms, potentially skewing results.

Although the proportion of people with high environmental commitment (environmental activists, *N* = 107; 5.6%) may be considered representative of the Spanish population, there is a significant imbalance in the number of participants within each group formed according to their level of environmental commitment. That complicates direct comparisons and limits the ability to draw robust conclusions about their specific attitudes or behaviors.

### 3.2 Practical implications

Talking about eco-anxiety instead of eco-worry contributes unintentionally to pathologizing essentially functional mechanisms. As much as we insist that eco-anxiety is not necessarily pathological, the mere fact of having to point it out confirms that intuitively, it has clear negative connotations in terms of mental health. In this respect, there is no justification to date for considering eco-anxiety a moral emotion, as proposed by Pihkala (2020), but it is possible to understand that a eco-worried citizen is a good citizen (Valentino et al., 2008).

Talking about eco-anxiety instead of eco-worry lays the foundation for individualized psychological treatment of problems with social roots that require political actions. Reducing everything to an intensely unpleasant individual experience weakens social forms of action. As Hickman et al. (2021) indicate if people associate “climate anxiety” with something excessive or irrational instead of a perceived inadequate government response to a severe global threat, the concept of eco-anxiety becomes inappropriate for guiding political decisions (Gregersen et al., 2024). In any case, if as suggested by Cunsolo et al. (2020), the urgent response needed to face the environmental crisis involves “*training for health professionals, enhanced clinical assessments, individual and group therapy strategies,”* among other clinical and individual actions it would be indicative of accepting social defeat and beginning to mitigate individual psychological impacts.

Talking about eco-anxiety instead of eco-worry may be creating an artificial scientific production with dubious practical utility. It leads to a dead end when it comes to promoting individual and collective pro-environmental actions because, in order to do what needs to be done, it will always be more reasonable to appeal to an increase in worry than to an increase in anxiety. The not-to-be-dismissed idea that anxiety and paralysis sometimes feed off each other is very poorly sold.

Talking about eco-anxiety instead of eco-worry can function as a dystopia that struggles for fulfillment. Eco-anxiety may be what researchers now anticipate that a significant part of the population will suffer when the reality of climate change is a succession of unquestionable catastrophes. However, for the moment, representative samples from several countries show that eco-anxiety, measured with the two most widely used instruments, the HEADS-13 and the CCAS, is very low, is more related to mental disorders than to well-being, and when it contributes to mobilizing pro-environmental behavior, it does so weakly (Innocenti et al., 2021; Whitmarsh et al., 2022; Wullenkord et al., 2021).

Our results showed that understanding the seriousness of the climate emergency problem, feeling and acting accordingly is something that, far from being associated with disorder and pathology, should be associated with well-being and satisfaction with life, at least for the moment and if we really seek to activate individual and collective behaviors that can mitigate and reverse the dire environmental and climatic crisis. All this without prejudice to the fact that eco-anxiety may be a specific response of a minority group of the population that probably requires clinical treatment and that will hardly be able to serve as a driving force for the necessary social changes.

## 4 Conclusion

We did not find high scores for eco-anxiety in any of the groups formed according to their level of commitment to the environment, nor where one might expect fertile ground for it, that is, in the activist environmentalist group, which, after all, was more aware of the seriousness of the situation and much more willing to act pro-environmentally under the restrictions and limitations that such awareness imposes. Nor do we find that the concept of eco-anxiety added value to that of eco-worry. Our results showed that, regardless of the level of prior environmental engagement, (1) Eco-worry, but not eco-anxiety, partially mediated the relationship between climate change perception and general willingness for environmental behavior, and (2) Eco-worry, but not eco-anxiety, connected with life satisfaction through the general willingness to behave pro-environmentally.

## Data Availability

The datasets presented in this study can be found in online repositories. The names of the repository/repositories and accession number(s) can be found: https://osf.io/6qn8y.
